# Metabolic Engineering of *Corynebacterium glutamicum* for Production of UDP-N-Acetylglucosamine

**DOI:** 10.3389/fbioe.2021.748510

**Published:** 2021-09-23

**Authors:** Rahul Gauttam, Christian K. Desiderato, Dušica Radoš, Hannes Link, Gerd M. Seibold, Bernhard J. Eikmanns

**Affiliations:** ^1^ Institute of Microbiology and Biotechnology, University of Ulm, Ulm, Germany; ^2^ Max Planck Institute for Terrestrial Microbiology, Marburg, Germany

**Keywords:** Corynebacterium glutamicum, metabolic engineering, activated amino sugars, sugar nucleotide, UDP-N-acetylglucosamine

## Abstract

Uridine diphosphate-N-acetylglucosamine (UDP-GlcNAc) is an acetylated amino sugar nucleotide that naturally serves as precursor in bacterial cell wall synthesis and is involved in prokaryotic and eukaryotic glycosylation reactions. UDP-GlcNAc finds application in various fields including the production of oligosaccharides and glycoproteins with therapeutic benefits. At present, nucleotide sugars are produced either chemically or *in vitro* by enzyme cascades. However, chemical synthesis is complex and non-economical, and *in vitro* synthesis requires costly substrates and often purified enzymes. A promising alternative is the microbial production of nucleotide sugars from cheap substrates. In this study, we aimed to engineer the non-pathogenic, Gram-positive soil bacterium *Corynebacterium glutamicum* as a host for UDP-GlcNAc production. The native *glmS*, *glmU*, and *glmM* genes and *glmM* of *Escherichia coli*, encoding the enzymes for UDP-GlcNAc synthesis from fructose-6-phosphate, were over-expressed in different combinations and from different plasmids in *C. glutamicum* GRS43, which lacks the glucosamine-6-phosphate deaminase gene (*nagB*) for glucosamine degradation. Over-expression of *glmS*, *glmU* and *glmM,* encoding glucosamine-6-phosphate synthase, the bifunctional glucosamine-1-phosphate acetyltransferase/N-acetyl glucosamine-1-phosphate uridyltransferase and phosphoglucosamine mutase, respectively, was confirmed using activity assays or immunoblot analysis. While the reference strain *C. glutamicum* GlcNCg1 with an empty plasmid in the exponential growth phase contained intracellularly only about 0.25 mM UDP-GlcNAc, the best engineered strain GlcNCg4 accumulated about 14 mM UDP-GlcNAc. The extracellular UDP-GlcNAc concentrations in the exponential growth phase did not exceed 2 mg/L. In the stationary phase, about 60 mg UDP-GlcNAc/L was observed extracellularly with strain GlcNCg4, indicating the potential of *C. glutamicum* to produce and to release the activated sugar into the culture medium. To our knowledge, the observed UDP-GlcNAc levels are the highest obtained with microbial hosts, emphasizing the potential of *C. glutamicum* as a suitable platform for activated sugar production.

## Introduction

Sugar nucleotides are activated forms of sugars and are composed of two moieties, a sugar and a nucleoside mono- or di-phosphate (Kleczkowski and Decker, 2015). Depending on the nucleoside phosphate attached, these sugar nucleotides are classified as nucleoside monophosphate sugars (such as cytidine monophospho-N-acetylneuraminic acid) or nucleoside diphosphate sugars, such as adenine- or uridine-diphosphate glucose, guanosine-diphosphate mannose or the activated amino sugar uridine-diphosphate-N-acetylglucosamine (UDP-GlcNAc) (Wagner et al., 2009). UDP-linked amino sugars have attracted a great deal of attention because of their indispensable role in cell wall biosynthesis and in the synthesis of natural glycoproteins, glycolipids, oligosaccharides, and glycosides by providing the sugar moieties (Barreteau et al., 2008; Thibodeaux et al., 2008; De Bruyn et al., 2015; Mikkola, 2020). UDP-GlcNAc plays a pivotal role in several metabolic processes in both prokaryotes as well as eukaryotes. In bacteria, UDP-GlcNAc serves as a precursor of cell wall components (Mengin-Lecreulx and van Heijenoort, 1993; Brown et al., 1994). In eukaryotes, it plays an important role in the synthesis of physiologically relevant glycoconjugates such as precursors for cell wall chitin, extracellular matrix polymers and bioactive glycoproteins (Moussian, 2008; Bond and Hanover, 2015; Corfield and Berry, 2015). In biotechnology, UDP-GlcNAc and other UDP-linked amino sugars serve as sugar donors for the chemical, enzymatic and/or microbial synthesis of a variety of native and non-native oligo- and polysaccharides for clinical (Thibodeaux et al., 2008) and medical (Wagner et al., 2009) purposes and for pharmaceutically relevant glycoproteins (Mueller et al., 2018; Natarajan et al., 2020).

**FIGURE 1:**
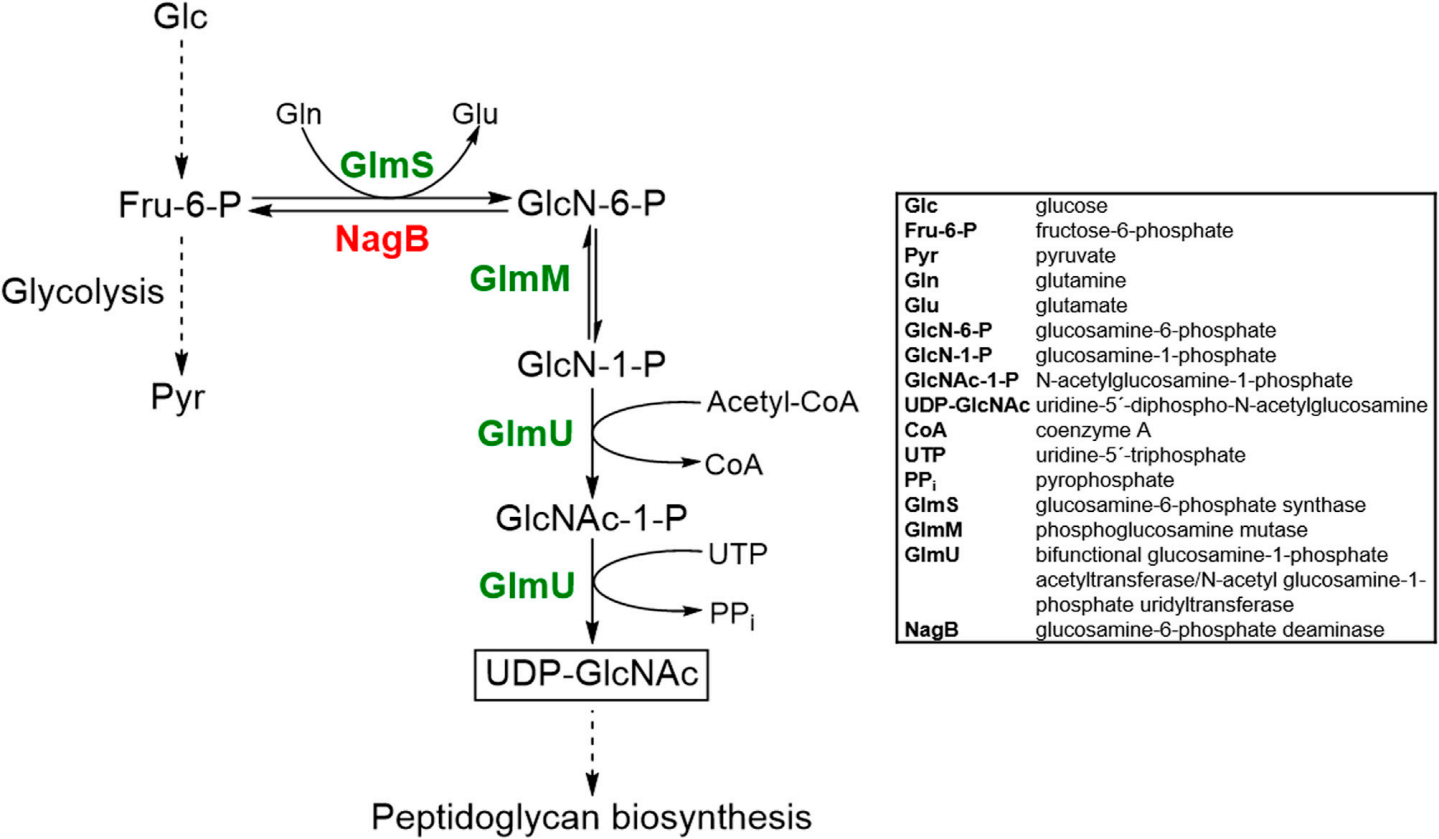
Schematic overview of the metabolic pathway responsible for UDP-GlcNAc biosynthesis in *C. glutamicum* and metabolic engineering strategies implemented to overproduce UDP-GlcNAc. Abbreviations are given in the box. The desired product UDP-GlcNAc is boxed. Product of genes that are knocked out or over-produced are shown in red or green text, respectively.

The UDP-GlcNAc biosynthetic pathway has been extensively elucidated in prokaryotes (Mikušová et al., 2000; Wang and Quinn, 2010; Li et al., 2012). UDP-GlcNAc is synthesized from fructose-6-phosphate (Fru-6-P) by three enzymes in four sequential enzymatic reactions (Figure 1). Firstly, Fru-6-P is converted to glucosamine-6-phosphate (GlcN-6-P) by glucosamine-6-phosphate synthase (GlmS). This reaction involves hydrolysis of L-glutamine to L-glutamate and ammonia, catalyzed by the N-terminal domain of GlmS, whereas the C-terminal domain is responsible for GlcN-6-P formation by utilizing the released ammonia (Durand et al., 2008). Secondly, GlcN-6-P is converted to glucosamine-1-phosphate (GlcN-1-P). This step is catalyzed by phosphoglucosamine mutase (GlmM) in a phosphorylated state in a ping-pong bi-mechanism involving glucosamine-1,6-bisphosphate as an intermediate (Jolly et al., 1999). The last two reactions, i.e., acetylation of GlcN-1-P to form GlcNAc-1-P and conversion of GlcNAc-1-P (plus UTP) to UDP-GlcNAc (plus pyrophosphate) are catalyzed by the bifunctional glucosamine-1-phosphate acetyltransferase/N-acetyl glucosamine-1-phosphate uridyltransferase GlmU (Mengin-Lecreulx and van Heijenoort, 1993; Mengin-Lecreulx and van Heijenoort, 1996; Milewski et al., 2006). The C-terminal GlmU domain is responsible for the acetylation, whereas the N-terminal domain catalyzes the uridylation reaction (Mengin-Lecreulx and van Heijenoort, 1994).

*C. glutamicum* is a non-pathogenic, aerobic, Gram-positive, biotin-auxotrophic soil bacterium that was initially described to be a natural producer of L-glutamate (Kinoshita et al., 1957). Since its discovery, the most appreciated application for this organism is L-glutamate and L-lysine production (Eggeling and Bott, 2015), however, the organism has also been employed for the production of other industrially relevant amino acids such as L-methionine, L-threonine, L-valine, L-tryptophan, phenylalanine, and isoleucine (Becker and Whittmann, 2015; Eggeling and Bott, 2015; Wendisch et al., 2016). The ease of cultivation of *C. glutamicum* combined with the generally regarded as safe (GRAS) status and robustness towards environmental stress contributed to the success of this bacterium in biotechnology. Furthermore, the utility of *C. glutamicum* in the production of a diverse range of bioproducts can be attributed to the sophisticated understanding of metabolic pathways regulation and the availability of advanced metabolic engineering strategies (Becker et al., 2016; Baritugo et al., 2018). Another advantageous feature of this organism is its ability to use a variety of sugars and to co-utilize different substrates (Blombach and Seibold, 2010; Wendisch et al., 2016). Owing to these advantageous traits, *C. glutamicum* has been engineered to produce numerous bioproducts other than amino acids, including isobutanol (Blombach et al., 2011), diamines (Schneider and Wendisch, 2011), polyhydroxybutyrate (Jo et al., 2006), 1,2-propanediol (Niimi et al., 2011), lactate (Okino et al., 2008), ethanol (Inui et al., 2004), cadaverine (Mimitsuka et al., 2007), xylitol (Sasaki et al., 2010) and GDP-L-fucose (Chin et al., 2013). Recently, it has been shown that metabolically engineered strains of *C. glutamicum* are also able to produce hyaluronic acid (Cheng et al., 2019a) and chondroitin (Cheng et al., 2019b), two products that include activated sugars (UDP-Glc, UDP-GlcNAc) in their synthesis.

The present study demonstrates for the first time the potential of *C. glutamicum* to intra- and extracellularly accumulate the UDP-linked activated amino sugar UDP-GlcNAc by modifying the biosynthetic pathway using metabolic engineering strategies. The employed enzymatic approach for *in vivo* UDP-GlcNAc biosynthesis in *C. glutamicum* provides a promising economical alternative to the complex chemical route and also provides the room for scaling up the production process to larger amounts.

## Material and Methods

### Bacterial Strains, Plasmids, and Culture Conditions

Bacterial strains and plasmids used in this study are listed in Table 1. For recombinant plasmid construction, genomic DNA of *E. coli* and of *C. glutamicum* was isolated and used as the template for specific gene amplification. *E. coli* DH5α was used for gene cloning.

**TABLE 1:** Strains and plasmids used in this study.

Strain or plasmid	Relevant characteristics	Source/References
**Strains**		
*E. coli* DH5α	F^−^ *φ*80*lacZ*∆M15 ∆(*lacZYA-argF*) U169 *endA1 recA1 hsdR17* (r_k_^−^, m_k_^+^) *supE44 thi* ^*−1*^ *gyrA996 relA1 phoA*	Hanahan (1983)
*E. coli* BL21	ompT *hsd*SB (r_B_-m_B_) *gal dcm* (DE3)	Studier (1990)
*E. coli BL21* (*pET28a- His6glmU*_*cg*_)	*E. coli* BL21 carrying the coding sequence for N-terminally His-tagged GlmU from *C. glutamicum* in vector pET28a	This study
*C. glutamicum* GRS43	*C. glutamicum* GRS with in-frame deletion of *cg2925-2,943*	Unthan et al. (2015)
*C. glutamicum* GlcNCg1	strain GRS43 carrying pCLTon1; Kan^R^	This study
*C. glutamicum* GlcNCg2	strain GRS43 carrying pCLTon1-*glmUSM*_*Cg*_; Kan^R^	This study
*C. glutamicum* GlcNCg3	strain GRS43 carrying pCLTon1-*glmUSM*_*Cg*_ and pRG_Duet1; Kan^R^ + Cm^R^	This study
*C. glutamicum* GlcNCg4	strain GRS43 carrying pCLTon1-*glmUSM*_*Cg*_ and pRG_Duet1-*glmM*_*Eco*_; Kan^R^ + Cm^R^	This study
*C. glutamicum* GlcNCg5	strain GRS43 carrying pCLTon1-*glmM*_*Eco*_ *US*_*Cg*_; Kan^R^	This study
*C. glutamicum* GlcNCg6	strain GRS43 carrying pCLTon1-*glmM*_*Eco*_ *US*_*Cg*_ and pRG_Duet1; Kan^R^ + Cm^R^	This study
*C. glutamicum* GlcNCg7	strain GRS43 carrying pCLTon1-*glmM*_*Eco*_ *US*_*Cg*_ and pRG_Duet1-*glmM*_*Eco*_; Kan^R^ + Cm^R^	This study
*C. glutamicum* GlcNCg8	strain GRS43 carrying pRG_Duet1; Cm^R^	Gauttam et al. (2019)
*C. glutamicum* GlcNCg9	strain GRS43 carrying pRG_Duet1-*glmM*_*Eco*_; Cm^R^	This study
**Plasmids**		
pRG_Duet1	dual-inducible *E. coli/C. glutamicum* shuttle vector (*P*_*tac*_, *lacI* ^*Q*^ *,* OriV_*C. glut*_ (pCG1), OriV_*E.coli*_ (p15A), *P*_*tetR/tetA*_ *, tetR*); Cm^R^	Gauttam et al. (2019)
pCLTon1	*E. coli*/*C. glutamicum* shuttle vector (*P*_*tet*_, *tetR*); Kan^R^	Lausberg et al. (2012)
pCLTon1-*glmUSM*_*Cg*_	pCLTon1 vector carrying *glmU, glmS* and *glmM* derived from *C. glutamicum* downstream of the ATc-inducible promoter *P*_*tet*_; Kan^R^	This study
pCLTon1-*glmUS*_*Cg*_	pCLTOn1-*glmUSM*_*Cg*_ with removed coding sequence for *glmM*; Kan^R^	This study
pCLTon1-*glmM*_*Eco*_ *US*_*Cg*_	pCLTon1-*glmUS*_*Cg*_ carrying *glmM* coding sequence from *E. coli* upstream of *glmU*, controlled by *P*_*tet*_; Kan^R^	This study
pET28a	Kan^R^; bacterial expression vector with T7 promoter	Novagen
pET28a-His6*glmU*_cg_	pET28a vector carrying the *glmU* gene from *C. glutamicum* with N-terminal histidine tag sequence	This study
pJET1.2/blunt	Linearized cloning vector for use in *E. coli*; Amp^R^	CloneJET PCR Cloning Kit (Thermo Scientific)
pJET-*glmM*_*Eco*_	pJET1.2/blunt carrying *glmM* from *E. coli* with C-terminal histidine tag sequence; Amp^R^	This study
pRG_Duet1-*glmM*_*Eco*_	pRG_Duet1 carrying *glmM* from *E. coli* with C-terminal histidine tag sequence controlled by *P*_*tac*_; Cm^R^	This study

*E. coli* strains were cultivated aerobically in 2xTY complex medium (Green and Sambrook, 2012) at 37°C on a rotary shaker at 150 rpm. For *C. glutamicum* cultivation, a single colony picked from a freshly prepared 2xTY agar plate was inoculated in 5 ml 2xTY seed culture and grown for 8 h at 30°C on a rotary shaker at 120 rpm. This seed culture was then used to inoculate a 50 ml 2xTY pre-culture in a 500 ml baffled flask and aerobically grown on a rotary shaker under the same conditions. Cells of an overnight pre-culture were harvested by centrifugation (4,200 × g, 15 min at 4°C) and washed twice with 0.9% (w/v) NaCl before inoculating modified CgXII minimal medium (Eikmanns et al., 1991) to an optical density at 600 nm (OD_600_) of about 1.5. In CgXII medium, the cells were grown aerobically on a rotary shaker (120 rpm at 30°C) in 500 ml baffled Erlenmeyer flasks containing 50 ml medium. The growth of bacterial cultures was monitored spectrophotometrically by measuring OD_600_ in an Ultrospec 2,100 pro spectrophotometer (GE Healthcare Life Sciences, Freiburg, Germany). Glucose (2% w/v) was added to this media as a carbon source. Whenever appropriate, cultures were supplemented with antibiotics at the following concentration: for *E. coli*, kanamycin (50 µg/ml), chloramphenicol (34 µg/ml), ampicillin (100 µg/ml), and for *C. glutamicum*: kanamycin (50 µg/ml), chloramphenicol (7.5 µg/ml). Solid media for 2xTY agar plates was prepared by adding agar (18 g/L). Gene expression was induced by adding isopropyl-β-D-thiogalactopyranoside (IPTG; 1 mM) and/or anhydrotetracycline (ATc; 0.25 μg/ml) to the cultures when the OD_600_ reached 3 to 4. Samples were removed at specific time intervals for determination of enzyme activities, for Western blot analysis and for determining product concentration.

### DNA Manipulation and Transformation

Standard molecular biology procedures were used for DNA isolation, gel electrophoresis, gene cloning, *E. coli* competent cells preparation, and transformation (Green and Sambrook, 2012). Restriction enzymes, T4 DNA Ligase, CloneJet^TM^ PCR Cloning Kit, and alkaline phosphatase employed in this study were obtained from Thermo Fisher Scientific (Waltham MA, United States) and used following the instructions from the manufacturer. Oligonucleotides used for gene amplification were ordered from biomers.net (Ulm, Germany) and are listed in Supplementary Table S1.

Coding sequences were first cloned into a subcloning vector (pJET1.2/blunt) and then into the respective expression vectors. Constructs were sequence-verified at Eurofins Genomics (Ebersberg, Germany). Polymerase chain reaction (PCR) conditions were optimized for each primer pair, and DNA fragments were amplified using Q5 High-Fidelity DNA polymerase (New England Biolabs). PCR products were separated by electrophoresis in agarose gels (1%; w/v) and purified using the NucleoSpin DNA extraction kit from Macherey and Nagel (Düren, Germany). Gibson Assembly Master^®^ Mix (New England Biolabs, Ipswich, MA, United States) was used to assemble fragments to generate expression cassettes following the instructions from the manufacturer. The primers were designed to incorporate 15–20 bp overlaps using the web-based “NEBuilderHiFi assembly tool” (New England Biolabs). The recombinant plasmids were isolated from *E. coli* transformants using the NucleoSpin plasmid purification kit from Macherey and Nagel, following the instructions of the manufacturer. Basic bioinformatic tools and software were used for designing oligonucleotides (Clone Manager v.7) and genome analysis (NCBI Blast).

For transformation, electrocompetent cells of *E. coli* DH5α and of *C. glutamicum* were prepared according to Dower et al., 1988, transformation of both organisms with plasmids was performed by electroporation using a MicroPulser Electroporator (Bio-Rad Laboratories GmbH, München, Germany) at 2.5 kV with 600 Ω resistance, as described before (Dower et al., 1988; Van der Rest et al., 1999). Recombinant strains (transformants) were selected on 2xTY agar plates containing respective antibiotics.

### Plasmid Construction

The *glmS, glmM,* and *glmU* genes, encoding glucosamine-6-phosphate synthase, phosphoglucosamine mutase and the bifunctional glucosamine-1-phosphate acetyltransferase/N-acetyl glucosamine-1-phosphate uridyltransferase, respectively, were amplified from *C. glutamicum* genomic DNA, using the primer pairs *glmS*_GIB-fwd/rev, *glmM*_GIB-fwd/rev, and *glmU_*GIB-fwd/rev, respectively. To increase the translational efficiency of *glmU*, the original TTG start codon of this gene was replaced by an ATG codon. All three fragments were joined in the order (*glmU* → *glmS* → *glmM*) by Q5^®^ Gibson assembly. The assembled fragments were then ligated into the *Sal*I*/Sac*I double-digested expression vector pCLTon1 (Lausberg et al., 2012), resulting in plasmid pCLTon1-*glmUSM*_*cg*_. Plasmid pCLTon1 carries the (anhydro) tetracycline- (ATc-) inducible promoter P_tet_ to control the expression of inserted genes. As host for the newly constructed plasmid, we chose *C. glutamicum* GRS43 (Unthan et al., 2015). This strain was transformed with vector pCLTon1 (empty vector as a control) and with plasmid pCLTon1-*glmUSM*_*cg*_ to generate the *C. glutamicum* strains GlcNCg1 (carrying pCLTon1) and GlcNCg2 (carrying pCLTon1-*glmUSM*_*cg*_).

For expression of the *E. coli glmM* gene in *C. glutamicum*, we replaced the corynebacterial *glmM* gene in pCLTon1-*glmUSM*_*cg*_ with the respective *glmM*_Eco_. For that purpose, an intermediate construct pCLTon1-*glmUS*_*Cg*_ was created by removing the coding region for *glmM*_*Cg*_ using *Bgl*II/*Spe*I restriction sites. The coding region for *glmM*_*Eco*_ was amplified from *E. coli* genomic DNA using *glmM*_*Eco*_-His6-fwd/rev primers and cloned into pJET1.2/blunt (a subcloning vector) to construct pJET-*glmM*_*Eco*_. After sequence verification, the *glmM*_*Eco*_ fragment was cut out using *Sbf*I and ligated into *Sbf*I-restricted pCLTon1-*glmUS*_*Cg*_ to construct pCLTon1-*glmM*_*Eco*_ *US*_*Cg*_. In addition, the *Sbf*I-cut-out *glmM*_*Eco*_ fragment was ligated into *SbfI-*restricted expression plasmid pRG_Duet1 (Gauttam et al., 2019), downstream of the IPTG-inducible promoter *P*_*tac*_ *,* to create pRG_Duet1-*glmM*_*Eco*_.

### Preparation of Cell Extracts

In order to detect proteins using Western blot and to determine intracellular enzyme activities, cells were harvested during the mid-exponential growth phase (OD_600_ of 10–12) by centrifugation (3,200 × g, 15 min, 4°C) and washed once with 0.9% NaCl. Cells were resuspended in 1 ml resuspension buffer (50 mM Tris-HCl, pH 7.5) and filled into 2 ml screw-cap tubes containing glass beads (250 µL). The tubes were then placed in a RiboLyser (Thermo Fisher Scientific, Heidelberg, Germany), and cells were disrupted three times using a pre-optimized program at a speed of 6.5 for 45 s with 5 min of intermittent cooling on ice to prevent protein denaturation due to frictional heat generation. Cell debris and glass beads were removed from the whole cell lysate by centrifugation (18,000 × g, 30 min, 4°C).

### Western Blot Analysis

To confirm *glmM* expression in cell-free extracts, the recombinant strains were grown in CgXII minimal medium, and cells were harvested at mid-exponential growth phase (OD_600_ of 10–12). Protein concentration was determined by employing a colorimetric Bradford assay using the Roti^®^-Nanoquant kit (Carl Roth, Karlsruhe, Germany). Prior to loading on the gel, the samples were mixed with SDS-PAGE loading dye [5-fold concentrated: 0.313 M Tris (pH 6.8), 200 mM dithiothreitol (DTT), 1% (w/v) SDS, 2% (w/v) glycerol, bromophenol blue (0.02%)], and boiled at 100°C for 10 min. The gels were prepared using TGX Stain-Free^TM^ FastCast^TM^ Acrylamide Kit (Bio-Rad Laboratories, Feldkirchen, Germany; Cat. #161-0,185). Cell extracts containing about 40 µg protein were loaded in each lane. Using the Trans-Blot Turbo^TM^ transfer system (Bio-Rad Laboratories, Cat. #170-4,272), protein bands were transferred to a PVDF membrane. This step was followed by overnight membrane blocking in 5% skimmed milk at 4°C with mild agitation. Next day, the membrane was washed (3× for 5 min) in Tris-buffered saline (pH 7.5) with Tween-20 (TBST), followed by 1 h incubation in a solution containing 6×-His Tag monoclonal antibody HIS.H8 (Thermo Scientific, Rockford, IL, Cat. #MA1-21315) at room temperature with mild agitation. After incubation with the primary antibody, the membrane was washed (3× for 5 min) again in TBST, followed by incubation with horseradish peroxidase (HRP)-labelled secondary antibody (Peroxidase AffiniPure Goat Anti-Mouse IgG, Cat. #115–035-003, Thermo Fischer Scientific, Darmstadt, Germany). Following this step, the membrane was washed again in TBST before being developed to visualize the bands using a SuperSignal^TM^ chemiluminescent substrate (Cat. #34095, Thermo Fischer Scientific) and an iBright Imaging system (Thermo Fischer Scientific).

### Enzyme Activity Assays

Glucosamine-6-phosphate synthase (GlmS) activity was determined spectrophotometrically using the glutamate dehydrogenase (GDH) based assay described by Badet et al. (1987). GDH catalyzes the reduction of 3-acetylpyridine adenine dinucleotide (APAD) into APADH that can be detected directly at 365 nm. The reaction mixture contained 50 mM potassium phosphate buffer, pH 7.5, 0.3 mM APAD, 10 mM fructose-6-phosphate, 6 mM glutamine, 50 mM KCl, GDH (3 U), cell extract (variable) in a total volume of 1 ml (Badet et al., 1987). Fructose-6-phosphate was added to initiate the reaction. To calculate the specific activity of GlmS in cell extracts, the millimolar extinction coefficient of reduced APADH at 363 nm (9.1 mM^−1^ cm^−1^) was used. One Unit (U) of activity is defined as the conversion of 1 μmole of glutamine to glutamate per minute.

Glucosamine-1-phosphate acetyltransferase/*N*-acetylglucosamine-1-phosphate uridyltransferase (GlmU) activity was determined using an enzymatic assay described previously by Mengin-Lecreulx and Van Heijenoort (1994). The assay involves the reaction between Ellman’s reagent and acetyl-CoA, resulting in an increase in absorbance at 412 nm. The reaction mixture contained 50 mM Tris-HCl, pH 7.5, 3 mM MgCl_2_, 1 mM acetyl-CoA, 1 mM 5,5-dithio-bis-2-nitrobenzoic acid (DTNB), cell extract (variable) in a total volume of 200 μL. The reaction was started by adding glucosamine-1-phosphate (1 mM). To calculate the specific activity of GlmU, the millimolar extinction coefficient of reduced DTNB at 412 nm (13.6 mM^−1^ cm^−1^) was used. One Unit (U) of activity is defined as the amount required to acetylate 1 μmole of glucosamine-1-phosphate per minute.

For determination of phosphoglucosamine mutase (GlmM) activity, we used a coupled enzyme assay described previously by Mengin-Lecreulx and van Heijenoort (1996), involving the GlmU reaction for determination of the glucosamine-1-phosphate production by GlmM. Purification of histidine-tagged *C. glutamicum* GlmU overproduced in *E. coli* BL21 (pET28a-His6*glmU*_cg_) and proof of activity of the purified enzyme is outlined in the Supplementary Material. The GlmM reaction mixture contained 50 mM Tris-HCl buffer pH 7.5, 3 mM MgCl_2_, 1 mM acetyl-CoA, 1 mM DTNB, purified GlmU (5 mg, 8.6 U/mg), cell extract (variable) in a total volume of 200 µL. The reaction was started by adding glucosamine-6-phosphate (1 mM).

### Uridine Diphosphate-N-Acetylglucosamine Quantification

To determine the total UDP-GlcNAc concentration in cells and culture broth, 100 μL of the culture was taken at a specified time point and mixed with 400 μL equimolar pre-cooled (at −20°C) acetonitrile: methanol mixture (50:50 v/v), followed by 30 min incubation at −20°C. Afterwards, cell debris was removed by centrifugation (13,000 rpm, 15 min, 4°C), and the supernatant (containing the cytosol and cell-free culture broth) was collected and stored at −80°C until further analysis.

To determine the extracellular UDP-GlcNAc concentration, 100 µL of the culture was sterile-filtered (0.2 µm), and the filtrate was quenched as described above. LC-MS/MS measurements were performed following the method previously described by Guder et al*.* (2017). For liquid chromatography (LC), an Agilent 1,290 Infinity II UHPLC system (Agilent Technologies) was used with iHILIC-Fusion(P) (50 × 2.1 mm, 5 µm) column at 30°C. An LC method previously described by Guder et al. (2017) with standardized runtime (2 min), flow rate (0.4 ml/min), injection volume (3 μL) and time between injections (0.5 min) was applied. LC-MS grade water with ammonium carbonate (10 mM) and ammonium hydroxide (0.2%) was used as LC solvent A. Acetonitrile (ACN) was used as LC solvent B and the gradient was 0 min 90% B; 1.3 min 40% B; 1.5 min 40% B; 1.7 min 90% B; 2 min 90% B. LC-treated samples were loaded onto an Agilent 6495 triple quadrupole mass spectrometer. The obtained LC-MS/MS data were converted into a text file using MSConvert (Chambers et al., 2012). Further data analysis was performed by an in-house software (Guder et al., 2017). Determination of absolute concentrations of UDP-GlcNAc was performed by using the ^13^C internal standard and authentic standards (Bennett et al., 2009; Guder et al., 2017).

To calculate the intracellular UDP-GlcNAc concentration, the value of the extracellular UDP-GlcNAc concentration was subtracted from the UDP-GlcNAc concentration of the whole culture broth, the resulting UDP-GlcNAc concentration was divided by the cell dry weight (CDW), that was calculated from the OD_600_ (using a ratio of 0.33 g CDW L^−1^ OD_600_^−1^, determined by weighing dried biomass from cultures with specific OD_600_ values). For the calculation of the intracellular UDP-GlcNAc concentration (mM), a specific cell volume of 1.95 µL/mg CDW was assumed (Gutmann et al., 1992). The intracellular UDP-GlcNAc level is also given in mg UDP-GlcNAc/g CDW to better compare extracellular and intracellular values. For that purpose, the UDP-GlcNAc concentration was multiplied with the molecular mass of UDP-GlcNAc (= 607.36 g/mol) and divided by g CDW/L. The extracellular concentration is given in mg UDP-GlcNAc/L by multiplying the measured extracellular concentration with the molecular mass of UDP-GlcNAc.

## Results

### Overexpression of Homologous Genes Encoding Enzymes of the Pathway for UDP-GlcNAc Synthesis in *C. glutamicum*

With the aim to engineer *C. glutamicum* for the production of the activated amino sugar UDP-GlcNAc, the native genes coding for enzymes involved in the respective pathway, namely *glmS* (Gene ID: 1020224), *glmM* (Gene ID: 1018587), and *glmU* (Gene ID: 1018935) were identified and employed for the construction of plasmid pCLTon1-*glmUSM*_*cg*_ (*Material and Methods* Section). The respective gene products are annotated as glucosamine-6-phosphate synthase GlmS, phosphoglucosamine mutase GlmM and bifunctional N-acetylglucosamine-1-phosphate uridyltransferase/glucosamine-1-phosphate acetyltransferase GlmU (Figure 1). As host for the newly constructed plasmid, we chose *C. glutamicum* GRS43, which has been generated as part of a genome-reduction project (Unthan et al., 2015) and lacks the *nagB* gene (*cg2928*) encoding glucosamine-6-phosphate deaminase (NagB) and thus, the enzyme for the backward reaction of GlmS (Uhde et al., 2013) (Figure 1).

**FIGURE 2:**
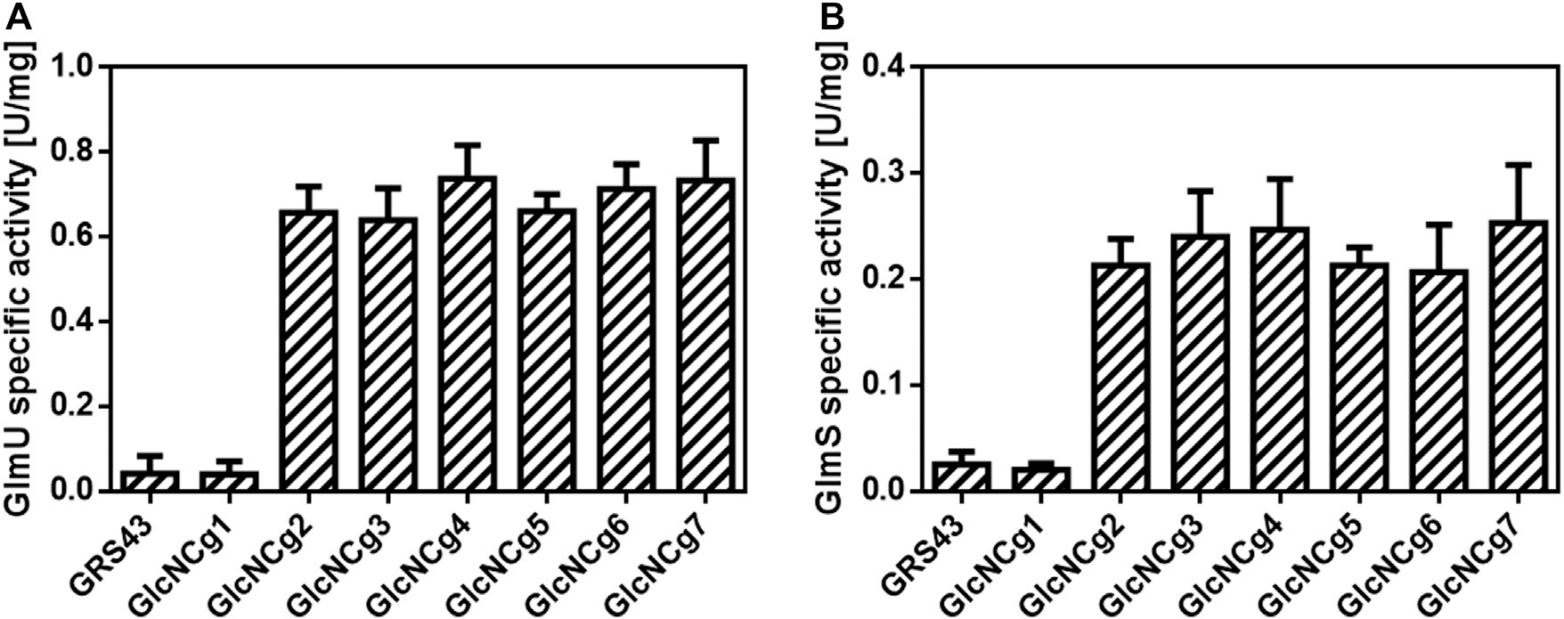
Specific GlmU **(A)** and GlmS **(B)** activities in cell extracts of *C. glutamicum* GRS43 and the recombinant strains *C. glutamicum* GlcNCg1 to GlcNCg7. For strain description, Table 1. Data represent mean values of triplicate assays from at least three individual cultivations. Error bars represent standard deviations (SD).

To test the newly constructed strain *C. glutamicum* GlcNCg2 and the control strains *C. glutamicum* GRS43 and GlcNCg1 (with empty plasmid pCLTon1) for (over)expression of *glmU, glmS* and *glmM*, they were cultured in CgXII minimal medium with glucose as carbon source and at an OD_600_ of about 4, plasmid-borne expression was induced by addition of ATc. The cells were harvested at the mid-exponential growth phase (OD_600_ of 10–12), and crude extracts for enzyme tests were prepared. As shown in Figure 2A, the specific GlmU activities were found to be about 16-fold higher in the extracts of strain GlcNCg2 (0.66 ± 0.04 U/mg) when compared to those in the extracts of control strains GRS43 and GlcNcg1 (0.04 ± 0.03 U/mg). Similar results were obtained for the GlmS activities, that were approximately 10-fold higher in the extracts of strain GlcNCg2 (0.21 ± 0.02 U/mg) in comparison to those observed in the extracts of strain GlcNcg1 (0.02 ± 0.01 U/mg). For so far unknown reasons, we were not able to detect GlmM activity in either of the cell extracts, indicating either that the assay used was not functional and/or not sensitive enough or that the plasmid-borne *glmM* gene is not expressed.

### Heterologous Expression of *E. coli glmM* in *C. glutamicum*

As described above, GlmM activity (and thus, *glmM* overexpression) could not be confirmed in cell extracts of *C. glutamicum* GlcNCg2, possibly due to very low (or no) expression of the native *C. glutamicum glmM.* To investigate this hypothesis and since GlmM from *E. coli* has been well characterized (Mengin-Lecreulx and van Heijenoort, 1996), we tested the heterologous expression of *glmM* from *E. coli* (*glmM*_*Eco*_) in *C. glutamicum*. For that purpose, we replaced the coding region for *glmM*_*Cg*_ in plasmid pCLTon1-*glmUSM*_*Cg*_ with the coding region for *glmM*_*Eco*_. The new construct pCLTon1-*glmM*_*Eco*_ *US*_*Cg*_ is different from pCLTon1-*glmUSM*_*cg*_ in the sense that *glmM*_*Eco*_ was C-terminally fused with the coding sequence for a histidine- (His-) tag and placed at the first position in the expression cassette *glmM-glmU-glmS*. In addition to plasmid pCLTon1-*glmM*_*Eco*_ *US*_*Cg*_, we constructed the pCLTon1-compatible plasmid pRG_Duet1-*glmM*_*Eco*_, carrying *glmM*_*Eco*_ under control of the IPTG-inducible *tac* promoter P_tac_. Subsequently, *C. glutamicum* GRS43 was transformed with plasmids pCLTon1-*glmUSM*_*cg*_, pCLTon1-*glmM*_*Eco*_ *US*_*Cg*_, pRG_Duet1-*glmM*_*Eco*_ and pRG_Duet1 in different combinations to construct *C. glutamicum* GlcNCg3, GlcNCg4, GlcNCg5, GlcNCg6, GlcNCg7, GlcNCg8, and GlcNCg9 (Table 1).

Enzyme assays were performed to confirm the overexpression of *glmU* and *glmS* in *C. glutamicum* GlcNCg3 to GlcNCg7. As shown in Figure 2A, the specific GlmU activities were found to be 15- to 18-fold higher in the extracts of strains GlcNCg3 to GlcNCg7, when compared to the specific activities in the extracts of reference strains GRS43 and GlcNCg1. In the same way, the specific GlmS activities were 11- to 15-fold higher in the extracts of strains GlcNCg3 to GlcNCg7 compared to those determined in the extracts of the control strains (Figure 2B).

**FIGURE 3:**
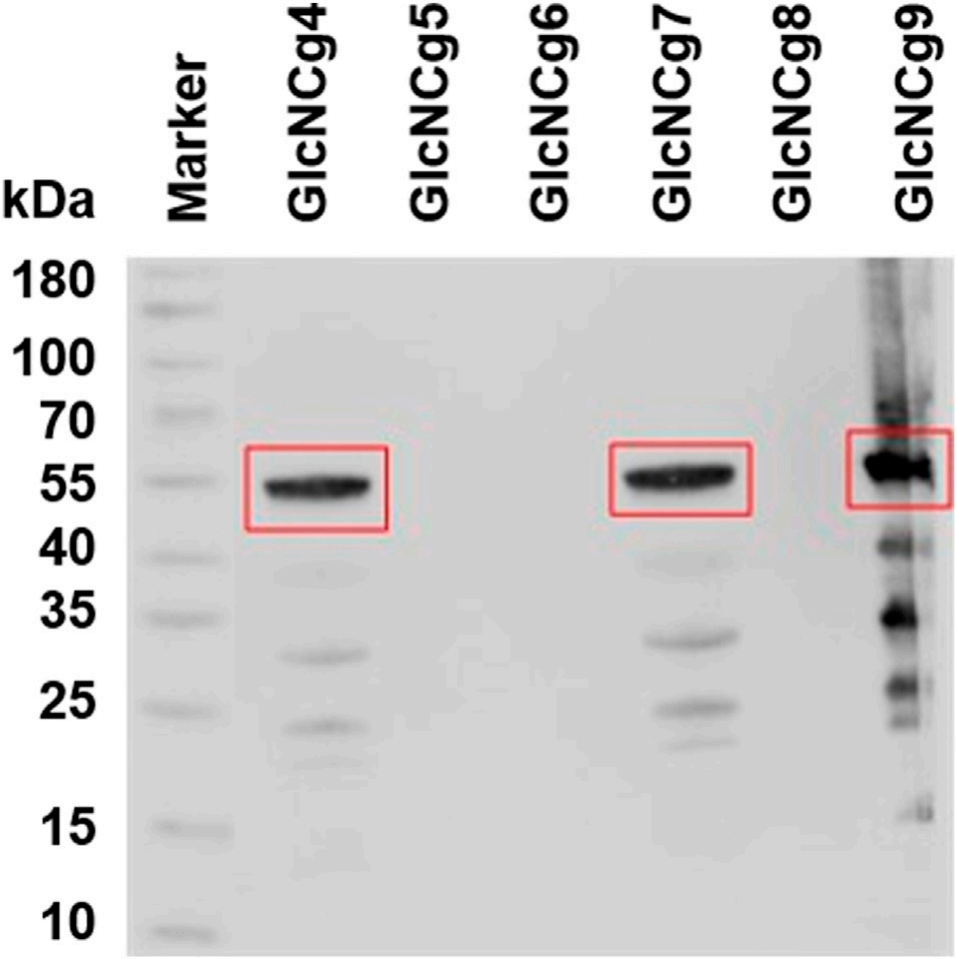
Western blot analysis of heterologous C-terminally His-tagged GlmM in cell extracts of recombinant *C. glutamicum* strains GRS43 and GlcNCg4 to GlcNCg9 (for strain description Table 1). The GlmM_*Eco*_ protein (calculated mass ∼49 kDa) is highlighted with a red rectangle.

To prove the heterologous expression of *glmM*_*Eco*_ and the presence of GlmM_*Eco*_ protein in the different *C. glutamicum* strains carrying pCLTon1-*glmM*_*Eco*_ *US*_*Cg*_ and/or pRG_Duet1-*glmM*_*Eco*_, Western blot analysis was performed with cell extracts of *C. glutamicum* GlcNCg4 to GlcNCg9, using the 6x-His-tag monoclonal antibody HIS.H8. As shown in Figure 3, a dense band well corresponding to the size of GlmM polypeptide (∼49 kDa) from *E. coli* was detected in the extracts of strains GlcNCg4, GlcNCg7, and GlcNCg9. As expected, GlmM_*Eco*_ protein was not detected in cell extracts of *C. glutamicum* GlcNCg8. However, the GlmM protein was also not detected in *C. glutamicum* GlcNCg5 and GlcNCg6 (Figure 3). Based on this observation, we hypothesize that ATc-inducible promoter *P*_*tet*_ is not strong enough to drive the expression of *glmM* in *C. glutamicum*, irrespective of the source of origin. A previous attempt to detect the production of His-tagged GlmM from *C. glutamicum* using the expression plasmid pCLTon1 failed too, and we could not observe a band corresponding to GlmM by immunoblot analysis of respective *C. glutamicum* extracts (data not shown).

It is noteworthy to mention that out of all recombinant strains GlcNCg4 and GlcNCg7 were the only strains which thus were proven to express all three genes (*glmU, glmS,* and *glmM*) involved in the prokaryotic UDP-GlcNAc biosynthesis pathway.

### Intracellular and Extracellular Accumulation of UDP-GlcNAc

Overexpression of *glmS*, *glmM,* and *glmU* in different combinations or from different plasmids were shown in the previous section. LC-MS/MS was employed for the quantification of UDP-GlcNAc in cells of recombinant *C. glutamicum* strains GlcNCg1 to GlcNCg9. As shown in Table 2, cells of the reference strain *C. glutamicum* GlcNCg1 (GRS43 carrying empty pCLTon1) showed minimal intracellular UDP-GlcNAc levels of about 0.25 mM (corresponding to 0.29 mg UDP-GlcNAc/g CDW) and those of strain GlcNCg2 (carrying pCLTon1-*glmUSM*_*cg*_) about 1.47 mM (i.e., 1.68 mg UDP-GlcNAc/g CDW). These results suggested that plasmid-based overexpression of *glmU* and *glmS* is sufficient for a significant increase of the UDP-GlcNAc content within the cells. Cells of *C. glutamicum* strain GlcNCg5 (carrying pCLTon1-*glmM*_*Eco*_ *US*_*Cg*_) contained about 1.51 mM (i.e., 1.68 mg UDP-GlcNAc/g CDW) and thus, comparable concentrations as strain GlcNCg2. These are the strains which showed overexpression of *glmU* and *glmS,* but not of *glmM*_*Cg*_ or *glmM*_*Ec*_. Surprisingly, an up to fourfold increase in UDP-GlcNAc levels were observed in *C. glutamicum* GlcNCg3 (5.85 mM) and GlcNCg6 cells (5.27 mM), carrying the same vectors as strains GlcNCg2 and GlcNCg5, respectively, but in addition the empty plasmid pRG_Duet1 (Table 2). The higher UDP-GlcNAc levels in *C. glutamicum* GlcNCg3 and GlcNCg6 correlate to an about twofold lower growth rate of these strains (μ = 0.16 to 0.17 h^−1^), when compared to their respective single-plasmid counterparts (GlcNCg2 and GlcNCg5; μ = 0.31 to 0.32 h^−1^) (Table 2). Maximal intracellular UDP-GlcNAc levels were observed in *C. glutamicum* GlcNCg4, carrying pCLTon1-*glmUSM*_*Cg*_ + pRG_Duet1-*glmM*_*Eco*_ (14.15 mM, i.e. 16.75 mg UDP-GlcNAc/g CDW) and GlcNCg7, carrying pCLTon1-*glmM*_*Eco*_ *US*_*Cg*_ + pRG_Duet1-*glmM*_*Eco*_ (13.06 mM, i.e. 15.69 mg UDP-GlcNAc/g CDW) (Table 2). These UDP-GlcNAc levels were around 55-fold higher than that of the reference strain GlcNCg1. The increased UDP-GlcNAc levels in strains GlcNCg4 and GlcNCg7 (μ = 0.18 ± 0.02 h^−1^) can be explained by the cumulative effect of reduced growth rate (μ = 0.17 to 0.18 h^−1^ vs 0.36 h^−1^) and proven overexpression of *glmUS*_*Cg*_ from plasmid pCLTon1 and of *glmM*_*Eco*_ from plasmid pRG_Duet1 (see above). Intracellular UDP-GlcNAc levels in *C. glutamicum* GlcNCg8, carrying empty plasmid pRG_Duet1 and showing also a relatively low growth rate of 0.23 h^−1^, were nearly as high as that of strain GlcNCg2 (1.26 vs 1.47 mM) (Table 2), although the former strain does not carry any plasmid-borne *glm* gene. This result indicates that a reduced growth rate is in favour of UDP-GlcNAc accumulation. *C. glutamicum* GlcNCg9 (carrying pRG_Duet1-*glmM*_*Eco*_) also showed a relatively low growth rate of about 0.23 ± 0.03 h^−1^ and only slightly higher intracellular UDP-GlcNAc concentration (about 1.55 mM) than strain GlcNCg8, indicating that *glmM*_*Eco*_ expression alone in combination with the reduced growth rate is not sufficient to substantially increase the intracellular UDP-GlcNAc levels.

**TABLE 2:** Growth rate, cell dry weight (CDW) at time of harvest and intracellular UDP-GlcNAc accumulation of recombinant *C. glutamicum* strains grown in minimal medium plus glucose and harvested in the exponential growth phase at an OD_600_ of about 10.

C. glutamicum strain	Growth rate (h^−1^)	g CDW/L	mg UDP-GlcNAc/g CDW	mM UDP-GlcNAc
GlcNCg1	0.36 ± 0.01	3.93 ± 0.05	0.29 ± 0.10	0.25 ± 0.08
GlcNCg2	0.32 ± 0.01	4.51 ± 1.73	1.68 ± 0.66	1.47 ± 0.22
GlcNCg3	0.16 ± 0.02	3.53 ± 0.20	6.92 ± 1.80	5.85 ± 1.52
GlcNCg4	0.17 ± 0.03	3.50 ± 0.10	16.75 ± 0.82	14.15 ± 0.07
GlcNCg5	0.31 ± 0.01	4.06 ± 1.60	1.68 ± 0.66	1.51 ± 0.28
GlcNCg6	0.17 ± 0.01	3.68 ± 1.45	5.49 ± 2.61	5.27 ± 1.98
GlcNCg7	0.18 ± 0.02	4.01 ± 1.52	15.69 ± 6.13	13.06 ± 1.62
GlcNCg8	0.23 ± 0.03	5.36 ± 2.15	1.49 ± 0.65	1.26 ± 0.30
GlcNCg9	0.20 ± 0.03	3.91 ± 1.48	1.84 ± 0.76	1.55 ± 0.30

Values are averages ±SD of three to six independent experiments (biological replicates).

**FIGURE 4:**
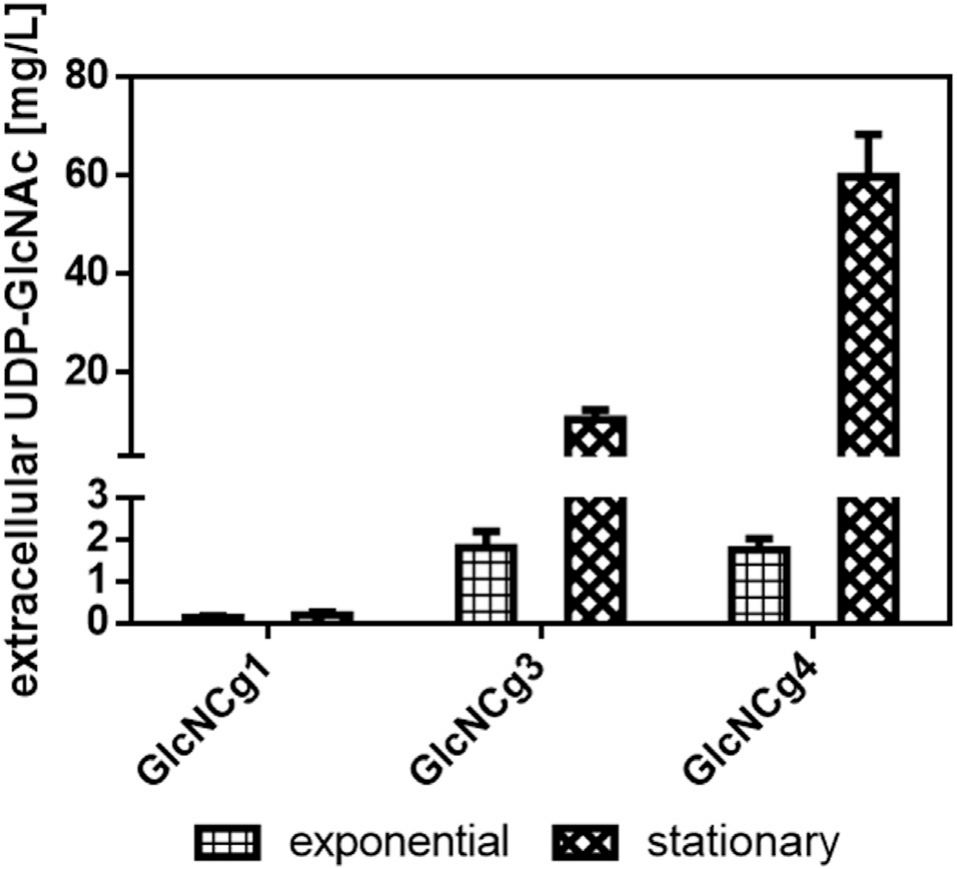
Extracellular accumulation of UDP-GlcNAc in *C. glutamicum* GlcNCg1, GlcNCg3 and GlcNCg4. For strain description Table 1. The cells were cultured in CgXII minimal medium with 2% glucose supplemented with inducers (1 mM IPTG, 250 ng/ml ATc). Data represents mean values of triplicate (exponential phase) or duplicate (stationary phase) assays from individual cultivations and error bars represent deviations.

To test also for extracellular UDP-GlcNAc accumulation, we analyzed the supernatants of *C. glutamicum* GlcNCg1 (basal intracellular UDP-GlcNAc level), *C. glutamicum* GlcNCg3 (elevated intracellular UDP-GlcNAc level), and *C. glutamicum* GlcNCg4 (highest intracellular UDP-GlcNAc level) cultures in the exponential (7 h after inoculation) and in the stationary growth phase (24 h after inoculation). In the exponential phase, the original host strain *C. glutamicum* GlcNCg1 accumulated extracellularly 0.13 ± 0.03 mg UDP-GlcNAc/L (i.e., below 0.25 µM) whereas strains GlcNCg3 and GlcNCg4 accumulated 1.8 ± 0.4 mg and 1.74 ± 0.3 mg UDP-GlcNAc/L, corresponding to roughly 3 µM in the culture supernatant (Figure 4), which is orders of magnitude lower than that within the cells (5.85 and 14.15 mM, respectively; Table 2). In the stationary phase, *C. glutamicum* GlcNCg3 showed significantly elevated extracellular UDP-GlcNAc accumulation with 10.42 ± 1.93 mg UDP-GlcNAc/L, i.e., about 17 µM, whereas the intracellular UDP-GlcNAc concentration was about 0.4 mM. An even higher extracellular accumulation of 59.80 ± 8.69 mg UDP-GlcNAc/L (about 0.1 mM) was observed for *C. glutamicum* GlcNCg4 (Figure 4). The intracellular concentration in stationary phase cells of *C. glutamicum* GlcNCg4 was about 0.67 mM, which is about 20-fold lower than in the exponential phase, but still 6- to 7-fold higher than the extracellular concentration (0.1 mM). This latter observation might indicate that the secretion (or the release) of UDP-GlcNAc into the medium is the rate-limiting step for UDP-GlcNAc production.

## Discussion

UDP-linked amino sugars are difficult to synthesize, however, chemical, enzymatic and chemoenzymatic approaches have been explored (Koizumi et al., 1998; Ahmadipour and Miller 2017; Zamora et al., 2017; Ahmadipour et al., 2018; Ahmadipour et al., 2019; Mikkola, 2020). Chemical synthesis of activated carbohydrates is demanding and relies on specialized expertise in carbohydrate chemistry; however, due to the lack of alternative routes, it remains a popular choice to synthesize non-natural sugar nucleotides (e.g., fluorinated nucleotide sugars) (Wagner et al., 2009; Tedaldi et al., 2012), which may serve for the synthesis of modified oligosaccharides, as enzyme inhibitors and/or in diagnostics (Mikkola, 2020). In general, sugar nucleotides generated from chemical routes are often quite expensive due to low extraction yield, and attempts made to scale up the production proved to be non-economical or impractical (Heidlas et al., 1992; Timmons and Jakeman, 2007; Wagner et al., 2009). Moreover, chemical approaches also suffer from other major drawbacks such as lack of stereoselectivity, tedious purification processes, and a low space-time yield (Zhao et al., 2010). Chemoenzymatic syntheses use biotransformation steps together with simple chemical steps, thus improving efficiency and enabling the synthesis of chemically defined nucleotide-activated sugars (Zamora et al., 2017). Using glucosamine and ATP as substrates and hexokinase, GlmM, and GlmU, Heidlas et al. (1992) firstly showed the enzymatic synthesis of UDP-GlcNAc. Further multi-enzyme cascades coupled with regeneration systems were developed (e.g., Elling, 1997; Bülter and Elling, 1999; Wahl et al., 2016). Efficient enzymatic synthesis of UDP-GlcNAc and other nucleotide sugars has recently been described in an up to 200 ml lab scale via enzyme cascades in repetitive batch mode (Fischöder et al., 2019). Compared to the chemical synthesis, this enzymatic process seems to be quite competitive, however, all these processes often require expensive starting materials (e.g., ATP, UDP and UTP), purified enzymes and complex operations for scaling up (Mikkola, 2020; Zhao et al., 2021), and therefore, are expensive and difficult to be used for industrial production.

To address above mentioned limitations and to make amino sugars (such as UDP-GlcNAc and UDP-GalNAc) available in large scale and in a cost-effective and sustainable manner, microbial production represents an interesting alternative to traditional chemical, enzymatic and chemoenzymatic routes. However, the use of microorganisms for the production of activated amino sugars relies heavily on a sound understanding of the microbial sugar biosynthetic pathways (Thibodeaux et al., 2008). Tabata et al. (2000) were the first to set up a microbial production system for UDP-GlcNAc, using a combination of six recombinant *E. coli* strains and *Corynebacterium ammoniagenes* DN510. In a rather complicated high-density incubation process, including permeabilization of the cells, the six different *E. coli* strains synthesized GlcNAc-1-P from externally added glucosamine and acetate by overexpression of the GlmM, GlmU, glucokinase, acetate kinase, phosphotransacetylase, or pyrophosphatase genes, respectively, whereas the latter provided the UTP (required for the very last step of UDP-GlcNAc synthesis, Figure 1) from orotic acid. The final titer of UDP-GlcNAc was 11.4 mM; compared to the *C. glutamicum* system presented here, this is a 100-fold higher concentration. However, several recombinant strains have to be cultivated separately and permeabilized in a second step. Further, the addition of several substrates (orotic acid, fructose, glucosamine, and acetate) at high concentrations are required, whereas *C. glutamicum* is producing UDP-GlcNAc in a single cultivation step in minimal medium containing glucose as the sole carbon source. Moreover, due to the permeabilization of the *E. coli* and *C. ammoniagenes* cells, it is likely that the downstream processing of the *C. glutamicum* system is presumably much easier. However, it is obvious that improvement of the *C. glutamicum* system is still required to obtain higher UDP-GlcNAc titers. It is striking that the strains tested in this work showed satisfactory performance even under non-optimized, standard laboratory growth conditions, thus, in parallel with strain optimization, production process optimization is also envisaged to reach higher biotransformation efficiency and product titer.

Rodríguez-Díaz et al. (2012a) showed for the first time that GlmS, GlmM and GlmU are responsible for UDP-GlcNAc biosynthesis in *Lactobacilli*, and they also found UDP-GlcNAc synthesis to be tightly regulated in *Lactobacillus casei* BL23 (Rodríguez-Díaz et al., 2012b). However, the authors constructed a recombinant *L. casei* BL23 strain overexpressing the homologous GlmS and GlmM genes and showing in complex medium with 0.5% glucose an about fourfold higher intracellular UDP-GlcNAc pool (3.18 µMol g protein^−1^), when compared to the parental strain (0.82 µMol g protein^−1^) (Rodríguez-Díaz et al., 2012a). Although the authors obviously did not analyze the culture broth for extracellular UDP-GlcNAc, all these observations indicated that the activities of the GlmSMU enzymes are sufficient for UDP-GlcNAc biosynthesis in prokaryotes and suggesting that it might be easily possible to use prokaryotic systems to produce UDP-GlcNAc from carbohydrates. In fact, we here were able to metabolically engineer *C. glutamicum* for production of this sugar nucleotide from glucose and could show that overexpression of *glmU*, *glmS,* and *glmM*_*Eco*_ in *C. glutamicum* GRS43 led to up to 16.75 mg UDP-GlcNAc per g of CDW. This corresponds to 51.5 µmol UDP-GlcNAc per g protein and thus to an about 16-fold higher intracellular UDP-GlcNAc accumulation when compared to the recombinant *L. casei* BL23.

In the stationary phase, we detected up to 60 mg UDP-GlcNAc L^−1^ in the culture supernatant of *C. glutamicum* GlcNCg4, indicating that the organism is able to release the activated sugar into the medium. Dedicated nucleotide sugar transporters were identified in eukaryotic cells and are localized in the membrane of the Golgi apparatus, thus providing nucleotide sugars for glycosylation reactions in this organelle (Gerardy-Schahn et al., 2001; Orellana et al., 2016). However, to our knowledge, such transporters are not present in bacteria. The accumulation of nucleotide sugars in the culture supernatant most probably reduces the overall conversion of UDP-GlcNAc since the suitable glycosyltransferases and sugar acceptors are located within the cells and are absent in the culture medium. It remains unclear why and how *C. glutamicum* releases UDP-GlcNAc in the stationary phase into the medium and what mechanisms are behind it. The big difference between the intracellular and the extracellular UDP-GlcNAc concentrations, in particular in strains GlcNCg1 and GlcNCg3, argues against a simple diffusion process and is in favour of protein-mediated transport. It can be speculated that the intracellular accumulation of UDP-GlcNAc in the stationary phase leads to conditions that e.g. affect peptidoglycan synthesis and/or the membrane state and thus possibly activate a channel protein. Time-course studies on the release of UDP-GlcNAc from the exponential to the stationary growth phase and concomitantly on the intracellular UDP-GlcNAc concentrations might shed more light on the UDP-GlcNAC release mechanism. However, the extracellular UDP-GlcNAc accumulation in the stationary phase simplifies the subsequent downstream processing and contributes to cheap production of UDP-GlcNAc with *C. glutamicum* compared to conventional enzymatic or chemical synthesis.

## Conclusion

In this study, *C. glutamicum* was engineered for the first time to produce the activated amino sugar UDP-GlcNAc. Homologous overexpression of *glmU* and *glmS* and heterologous expression of the *E. coli glmM* from two plasmids increased the intracellular concentration of UDP-GlcNAc more than 50-fold in a range that is nearly 20-fold higher than that obtained with recombinant *L. casei* BL23 cells. In addition, we demonstrate the accumulation of up to 60 mg UDP-GlcNAc/L in culture supernatants of recombinant *C. glutamicum* strains, which simplifies downstream processing and may contribute to product stability. Our results provide valuable information to develop this organism as a promising alternative to cost-intensive chemical methods for industrial production of UDP-GlcNAc. Moreover, our results indicate the potential of *C. glutamicum* to produce nucleotide sugars and their derivatives.

## Data Availability

The original contributions presented in the study are included in the article/Supplementary Material, further inquiries can be directed to the corresponding author.

## Abbreviations

AmpR, ampicillin resistance; AMP, adenosine monophosphate; ATc, anhydrotetracycline; CMP, cytosine monophosphate; CmR, chloramphenicol resistance; IPTG, Isopropyl-β-D-thiogalactopyranoside; KanR, kanamycin resistance; UDP, uridine diphosphate; UDP-GlcNAc, UDP-N-acetylglucosamine
