# Supplementary material for: Metabolic Engineering of Corynebacterium glutamicum for Production of UDP-N-Acetylglucosamine
Source: Front Bioeng Biotechnol. 2021 Sep 23;9:748510. doi: 10.3389/fbioe.2021.748510 (PMC8495162; doi:10.3389/fbioe.2021.748510)
Supplement: Supplementary file 1 [file Table1.DOCX]

**Supplementary TABLE S1 |**  Oligonucleotides used in this study. Restriction sites are indicated in bold.

| **Oligonucleotides** | **Sequence (5’ → 3’)** |
| --- | --- |
| *glmU*_GIB-fwd | CTTGTTGATACACCTGCAGGTTCGAATAAGGAGACGTAATGAGCGCAA |
| *glmU*_GIB-rev | TGTACTCCTGCTTCGAATTAGCCTTCCTGGTTGTGGA |
| *glmS*_GIB-fwd | GGAAGGCTAATTCGAAGCAGGAGTACAATGCGCATGTGTGGAATTGT |
| *glmS*_GIB-rev | CTGACTCCTATTCAACGTTATTCGACGGTGACAGACTTTG |
| *glmM*_GIB-fwd | CGTCGAATAACGTTGAATAGGAGTCAGCATGACTCGACTATTTGGAAC |
| *glmM*_GIB-rev | ACGGCCAGTGAATTCGAGCTTTAGACTTCTGCAACCACTG |
| *glmM_Eco_*-His6-fwd | AAACCTGCAGG**GCGGCCGC**TAAGAAGGAGATATACCATGAGTAATCGTAAATATTTCG |
| *glmM_Eco_*-His6-rev | AAA**CCTGCAGG**GACGTCTTAATGATGATGATGATGATGAACGGCTTTTACTGCATCGGCG |

**Assay for phosphoglucosamine mutase (GlmM) activity**

To establish the GlmM activity assay, we purified the *C. glutamicum* GlmU protein (N-terminal histidine tagged GlmU_Cg_) expressed in *E. coli* (BL21), using the pET system (pET28a-His6*glmU_Cg_*) and following the method described previously (Mengin-Lecreulx and van Heijenoort, 1996). The functionality and stability of GlmU protein was checked by its GlmU activity, using Ellman’s reagent (refer to Material and Method). Variable amounts of substrates were tested to confirm that the substrates are not limiting in the reaction mixture, the optimal GlmU activity was observed at 1 mM acetyl-CoA and 1 mM glucosamine-1-phosphate. Unfortunately, for unknown reason all attempts to establish a functional assay for determining GlmM activity in cell extracts of *C. glutamicum* strains proved to be unsuccessful.
